# Carotidynia Versus Transient Perivascular Inflammation of the Carotid Artery (TIPIC) Syndrome: Finding Common Ground

**DOI:** 10.7759/cureus.17684

**Published:** 2021-09-03

**Authors:** Sheldon P Hersh, Perry Gerard, Joshua Hersh

**Affiliations:** 1 Otolaryngology, Lenox Hill Hospital, Forest Hills, USA; 2 Radiology, Westchester Medical Center, Valhalla, USA; 3 Neurology, Southern Ocean Medical Center, Manahawkin, USA

**Keywords:** unilateral neck pain, tipic syndrome, diagnostic tool, clinical entity, contentious term

## Abstract

Carotidynia remains mired in controversy. Whether to identify this self-limiting unilateral neck pain as a distinct clinical entity or a diagnostic sign associated with a variety of conditions remains a topic of ongoing debate. Adding to the discussion is the occasional finding on imaging studies of a transient inflammatory process surrounding the carotid artery in a number of individuals who present with unilateral neck pain. Although some use carotidynia as the designation of choice by which to identify this inflammatory process, the acronym TIPIC (transient perivascular inflammation of the carotid artery) syndrome is being touted as a far more descriptive and less contentious alternative. Having TIPIC syndrome replace carotidynia, however, need not necessarily signal the latter’s outright elimination as some have advocated. When used as a diagnostic sign, carotidynia provides an appreciation of the many conditions that may be associated with idiopathic unilateral neck pain.

## Introduction

Carotidynia, a term first introduced by Fay in 1927 [1], was so named to describe the pain and tenderness associated with the application of pressure on or about the area of the carotid bifurcation (Fay’s Sign). Although Fay had initially intended for carotidynia to serve as a diagnostic aid in cases of atypical facial neuralgia, its subsequent use in other contexts has given rise to differences of opinion as to how carotidynia should be identified. Fueling the discussion is whether carotidynia is to be recognized as a distinct clinical entity or as a diagnostic symptom commonly associated with an array of diverse conditions [2]. A consensus on how best to characterize carotidynia remains at an impasse.

Adding to the controversy is the relatively recent finding on both ultrasound and imaging studies of an enhancing amorphous tissue surrounding the carotid artery in select cases of idiopathic unilateral neck pain. A number of reports appearing in the literature have chosen carotidynia as the preferred designation by which to identify this amorphous tissue [3,4], a decision that has once again proven contentious. Calling carotidynia “confusing”, Lecler et al [5] coined the term TIPIC (Transient Perivascular Inflammation of the Carotid Artery) syndrome as a preferable alternative. Moreover, these authors have joined others [6] in calling for the term carotidynia to be removed entirely from use.

Despite their obvious differences, TIPIC syndrome and carotidynia need not be mutually exclusive. TIPIC syndrome communicates the presence of a highly distinctive, site-specific inflammatory process that displays both clinical and radiological consistency. Carotidynia, on the other hand, has been anything but consistent and would function best as a diagnostic tool much the way Fay had initially intended. Once so accepted and identified, carotidynia can work in concert with TIPIC syndrome and provide complementary perspectives on how best to approach idiopathic unilateral neck pain.

## Case presentation

A 74 y/o female presented with a 6- to 7-day history of persistent left neck pain that radiated to the ipsilateral face. When asked to identify where the pain was most intense, she pointed to an area bordering on the left carotid bifurcation as being exquisitely tender and painful. She was quick to mention that swallowing would also give rise to pain in the area in question. The patient denied any antecedent or concurrent symptoms relating to an upper respiratory infection or regional inflammatory process. She had remained afebrile from the time the pain first appeared. There was no past history of cervical manipulation, regional trauma, or surgical intervention involving either the neck or nearby structures. Her medical history was notable for Graves' Disease, seizure disorder, lumbar stenosis, and aortic valve replacement. The patient had been seen days earlier by her primary care physician who noted a “slightly red throat” and prescribed amoxicillin/clavulanate which reportedly failed to provide any relief. 

On physical examination, no swelling or asymmetry of the neck was visualized. Palpation of the neck resulted in an immediate and sharp increase in pain particularly when pressure was applied to the area adjoining the left carotid bifurcation. No adenopathy or neoplasia was identified nor bruit or thrill detected. The ears, nose and throat/head, and neck were otherwise clear. No crepitus was appreciated nor pain elicited upon palpating the temporomandibular joints. Flexible nasopharyngolaryngoscopy revealed no pathology.

**Figure 1:**
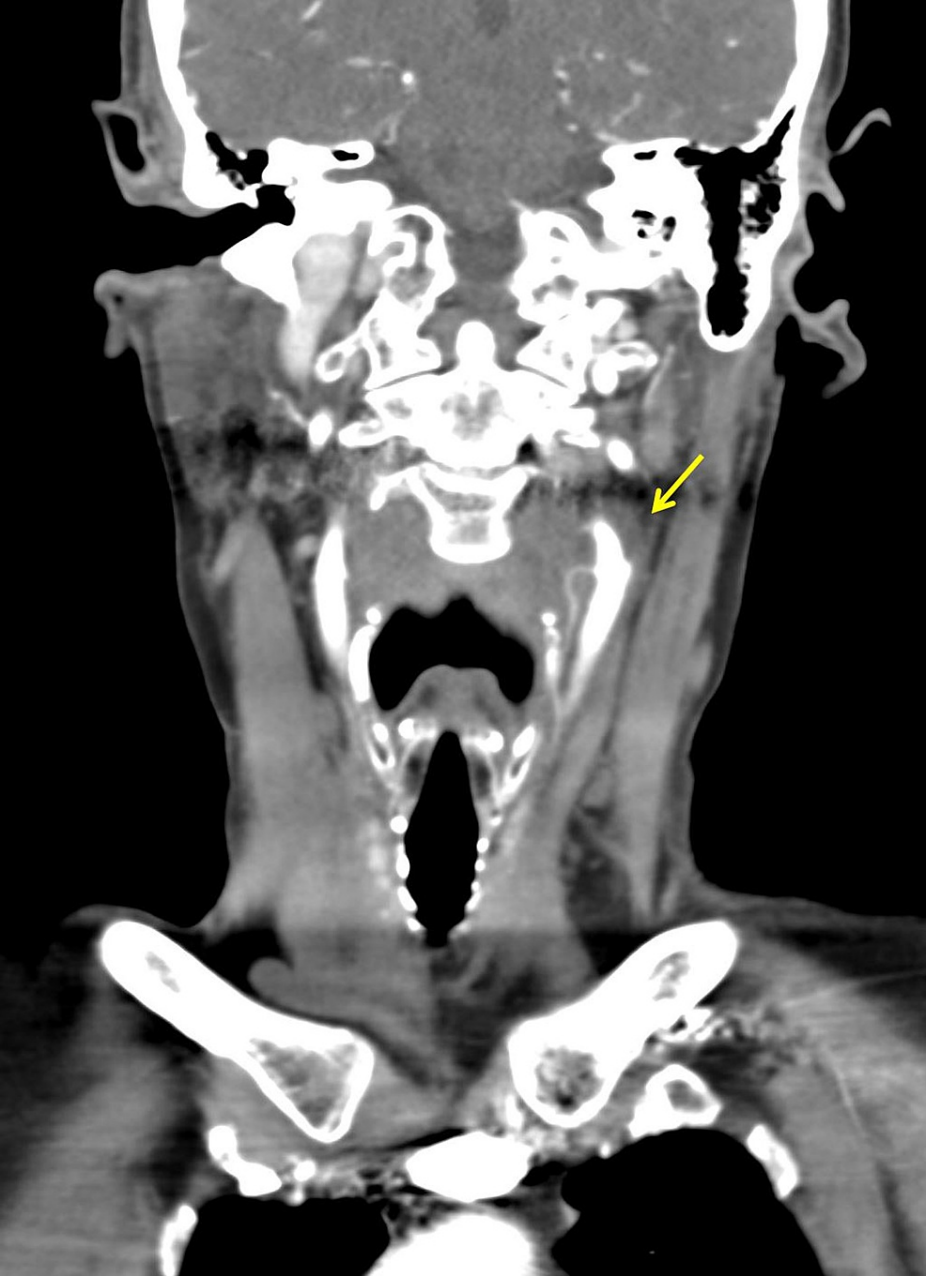
Coronal contrast-enhanced CT image demonstrating inflammatory soft tissue (arrow) surrounding the left distal common carotid trunk and carotid bifurcation without notable luminal narrowing.

**Figure 2:**
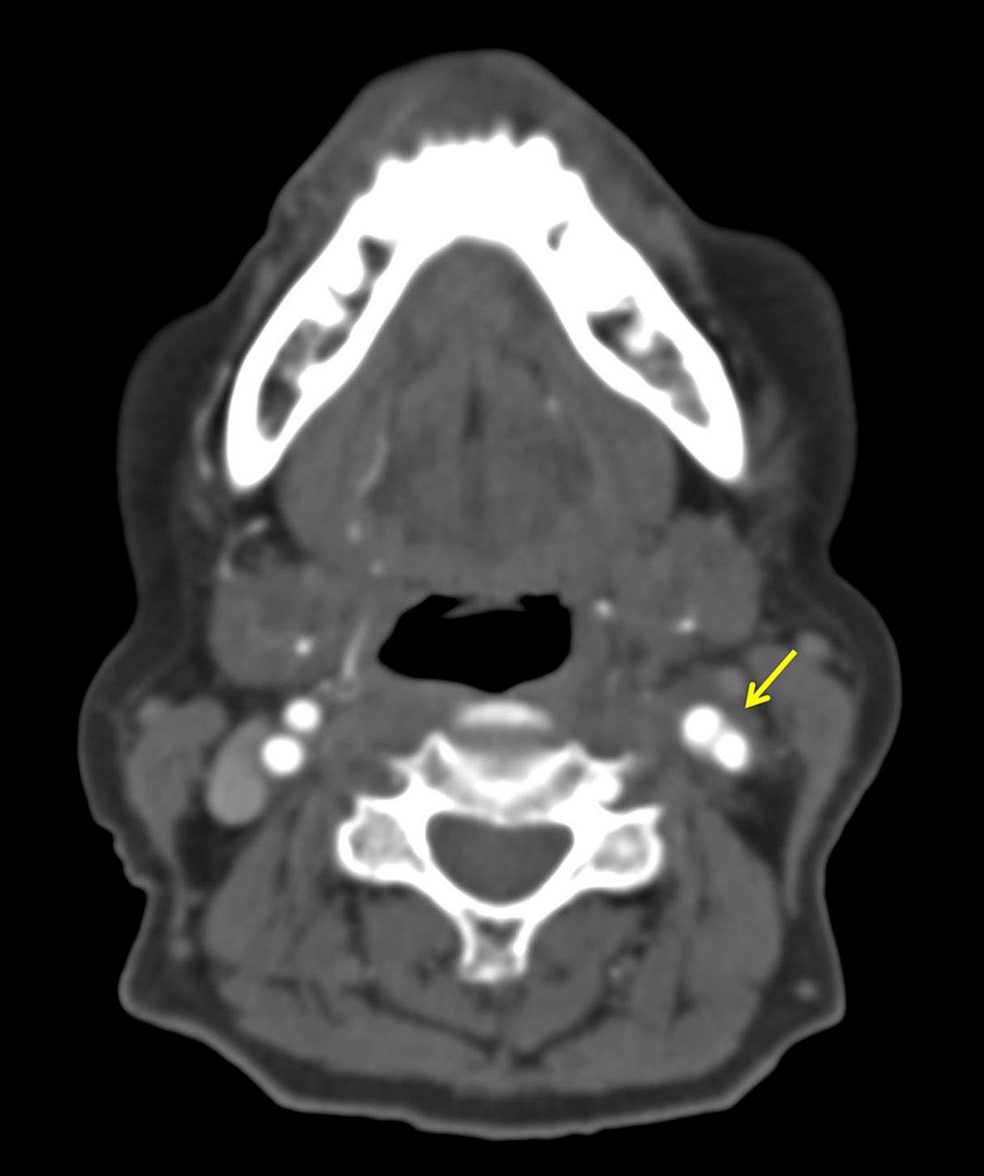
Axial contrast-enhanced CT image revealing the presence of enhancing inflammatory soft tissue (arrow) encircling the left carotid bifurcation.

In light of the location and unremitting nature of the pain, CT angiography was obtained and revealed the presence of an enhancing inflammatory tissue encircling the left common carotid artery and bifurcation. No evidence of luminal narrowing was noted (Figures 1, 2). The patient ultimately sought consultation elsewhere and was lost to follow-up.

## Discussion

The term carotidynia was first introduced by Fay in 1927 with the clear intention that it be used as a “diagnostic sign” by which to isolate possible “sites of involvement” in cases of atypical facial neuralgia [1]. Carotidynia, however, has since been assigned other, often conflicting identities, the nature of which continues to engender dispute. Whether to have carotidynia function as a diagnostic tool [2] or be classified as a migraine variant [7] or other distinct clinical entity [8] lies at the heart of the controversy. Deepening the debate is the opinion that carotidynia serves no practical purpose and should be removed entirely from use [5,6].

In its 1988 Classification of Headache Disorders [9], the International Headache Society (IHS) listed the following criteria needed to diagnose carotidynia: pain, swelling or increased pulsation upon applying pressure, appropriate investigations fail to reveal any structural abnormality, pain on the affected side of the neck radiating to the ipsilateral side of the head and pain lasting less than 2 weeks in duration.

The criteria set forth by the IHS in 1988, however, was seen as lacking the requisite specificity and consistency [6] needed to identify carotidynia as an independent entity. By 2004, the IHS overturned its earlier decision expunging carotidynia from its Classification of Headache Disorders [10]. Basing its decision on a review of the literature, the IHS noted that carotidynia could no longer be identified as a clinical entity but rather “a syndrome encompassing many varieties of pain in the carotid region.”

A wide range of both vascular and non-vascular disorders have been cited as possible sources of unilateral neck pain. Vascular disorders so implicated include aneurysm, carotid body tumor, dissection, occlusion, large vessel vasculitides and fibromuscular dysplasia [11]. Non-vascular causes are typically the direct result of inflammatory, infectious or neoplastic processes of the head and neck [4,11]. Amongst this later group are aphthous ulcers, dental disease, temporomandibular joint disease, migraine, superior laryngeal nerve neuralgia [12], elongated styloid process or ossification of the stylohyoid ligament (Eagle Syndrome) [13] and hyoid bone malformations [14].

Ultrasound and imaging studies have proven invaluable in helping to clarify many of the uncertainties that often arise in cases of idiopathic unilateral neck pain/carotidynia. These modalities not only assist in ruling out many of the diverse conditions known to cause unilateral neck pain but serve to identify the occasional presence of an enhancing amorphous soft tissue encircling the carotid artery at or near the bifurcation.

In a multicenter study Lecler et al [5] reported on 47 patients presenting with idiopathic neck pain centering about the carotid bifurcation. Ultrasound and imaging studies revealed a distinctive soft amorphous tissue surrounding the carotid bifurcation in all cases. Normal blood flow with little or no luminal compromise was visualized and the soft tissue in question proved self-limiting in nature generally resolving within 2 weeks.

Although earlier case reports refer to this inflammatory soft tissue as carotidynia, Lecler and colleagues argue that Transient Perivascular Inflammation of the Carotid Artery (TIPIC Syndrome) is a far more descriptive and less contentious designation and should therefore replace carotidynia [5]. They further advocate for including TIPIC syndrome in the International Classification of Headache disorders if four major criteria are met:

1) The presence of acute pain involving the carotid artery with possible radiation to the head

2) Eccentric PVI on imaging

3) The exclusion of other diagnostic entities on imaging

4) Resolution within a 2-week window occurring either spontaneously or with use of anti-inflammatory medication

TIPIC syndrome possesses the specificity and consistency that carotidynia so often lacks. It communicates a singularly unique clinical entity by virtue of its distinctive clinical presentation and the consistent radiographic finding of an inflammatory tissue surrounding the carotid trunk at or near the bifurcation. Given that much of the controversy surrounding carotidynia relates to its use as a distinct clinical entity, having it serve exclusively as a diagnostic sign, much the way Fay had initially intended, would prove to be a sensible compromise. When used in this manner, carotidynia provides an appreciation of the many conditions known to cause unilateral neck pain. The outright removal of carotidynia could well prove self-defeating.

In its preface to the second edition [10], the IHS stresses that “It is important for any field in medicine to have a generally accepted classification that is used throughout the world.” Such has not been the case with carotidynia. When viewed as a distinct clinical entity, carotidynia has shown itself to be consistently inconsistent and confusing. Carotidynia is much better suited to function as a diagnostic aid by providing the insight needed to appreciate the numerous conditions that are associated with idiopathic unilateral neck pain. TIPIC syndrome, on the other hand, exemplifies a distinct clinical entity owing to its specificity and consistency. Having carotidynia and TIPIC syndrome work in tandem will, most assuredly, provide an insightful guide on how best to approach idiopathic unilateral neck pain.

## Conclusions

Carotidynia continues to engender controversy. Although the term carotidynia was initially intended to serve as a diagnostic aid in cases of atypical facial neuralgia, its identity continues to vacillate between that of a diagnostic symptom commonly associated with an array of diverse conditions and a distinct clinical entity. How best to identify carotidynia remains a matter of dispute. The occasional finding on imaging studies of a transient inflammatory process surrounding the carotid artery in some individuals presenting with unilateral neck pain has furthered the debate. Some continue to use the term carotidynia as the designation of choice by which to identify this inflammatory process whereas others argue that carotidynia be removed and replaced with the acronym TIPIC (transient perivascular inflammation of the carotid artery) Syndrome. Carotidynia is much better suited to function as a diagnostic aid by providing an appreciation of the numerous conditions that have been shown to cause idiopathic unilateral neck pain. TIPIC syndrome, on the other hand, is far more descriptive and exemplifies a distinct clinical entity by reason of its specificity and consistency. Having carotidynia and TIPIC syndrome partner with one another will, most assuredly, provide a useful guide on how to approach the often-perplexing subject of idiopathic unilateral neck pain.
